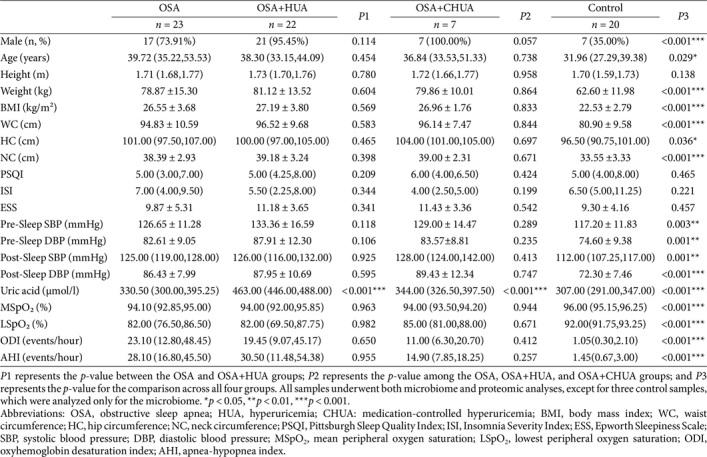# Association between Serum Uric Acid Levels and Salivary Microbiota in Patients with Obstructive Sleep Apnea

**DOI:** 10.4014/jmb.2503.03042

**Published:** 2025-06-23

**Authors:** Yujia Lu, Wanxin Zhang, Min Yu, Xuehui Chen, Chunyan Liu, Xuemei Gao

**Affiliations:** 1 Department of Orthodontics, Peking University School and Hospital of Stomatology, Beijing 100081, P.R. China; 2 Center for Oral Therapy of Sleep Apnea, Peking University Hospital of Stomatology, Beijing 100081, P.R. China; 3 Department of Orthodontics, Shanghai Ninth People's Hospital, Shanghai Jiao Tong University School of Medicine, Shanghai 200011, P.R. China; 4 Department of Orthodontics, Hebei Key Laboratory of Stomatology and Hebei Technology Innovation Center of Oral Health, School and Hospital of Stomatology, Hebei Medical University, Shijiazhuang 050017, P.R. China

**Keywords:** OSA, hyperuricemia, oral microbiome, 16S rRNA, Astral DIA

## Abstract

The microbiota is associated with obstructive sleep apnea (OSA) and hyperuricemia (HUA), but the relationship between oral microbiota and OSA-related HUA remains unclear. Our study investigated salivary microbiota differences between individuals with OSA and those with both OSA and HUA, and explored the link between oral microbiome alterations and uric acid fluctuations in OSA patients. Seventy-two adults were divided into four groups: controls (n = 20, 33.75 ± 9.46 years), OSA (n = 23, 44.08 ± 13.70 years), OSA with comorbid HUA (OSA+HUA, n = 22, 40.18 ± 9.58 years), and OSA with medication-controlled HUA (n = 7, 44.56 ± 15.14 years). Salivary microbiota and proteomic profiles were analyzed using 16S rRNA sequencing and Astral DIA. OSA and OSA+HUA showed reduced alpha-diversity compared to controls. The OSA+HUA group had increased *Oribacterium* abundance relative to the OSA group, which decreased after uric acid treatment, whereas *Rothia*, *Capnocytophaga*, and *Aggregatibacter* showed the opposite trend. 104 differentiated proteins were identified between the OSA and OSA+HUA groups. *Oribacterium* was positively correlated with several antioxidant proteins, while the other three genera were negatively correlated. This study identifies non-invasive biomarkers in the OSA+HUA group, as the first of its kind, highlighting the role of oral microbiota in future research and therapies.

## Introduction

Obstructive sleep apnea (OSA) is a disorder characterized by repeated episodes of apnea and hypoventilation during sleep [1]. It affects approximately 1 billion people worldwide [2]. The condition involves the frequent blockage of the upper airway, either fully or partially, during sleep. This blockage leads to arousal, reduced oxygen levels, and elevated carbon dioxide concentrations [3]. The resulting intermittent hypoxia is a major trigger for oxidative stress [4]. Prolonged intermittent hypoxia may lead to increased inflammatory responses and multisystem damage.

It is widely recognized that OSA is a heterogeneous disease consisting of multiple subtypes, characterized not only by the diversity of anatomical abnormalities, neuromodulatory disorders, and metabolic factors, but also by its association with a range of comorbidities, such as cardiovascular and metabolic diseases [5]. Hyperuricemia (HUA) is a metabolic condition primarily caused by increased synthesis or decreased elimination of serum uric acid. It may lead to gout, urate nephropathy, and urolithiasis, and is closely associated with OSA [6]. The severity of OSA is positively associated with serum uric acid levels [7]. Studies have shown that a 1 mg/dL rise in uric acid levels is associated with a 16% higher risk of OSA [8]. Many observational studies have shown that individuals with OSA tend to have higher uric acid levels than those without the condition [8–10], and these levels normalize following effective OSA treatment [11].

Uric acid represents the final metabolite in the breakdown of purines [12]. At physiological concentrations, it acts as a strong antioxidant [13]. It can effectively scavenge carbon-centered and peroxyl radicals under hydrophilic conditions [14], preventing reactive oxygen species (ROS)-induced endothelial cell injury [15, 16]. Uric acid can also react with peroxynitrite to form nitrated and nitrosated derivatives. These derivatives release nitric oxide (NO) and increase NO availability [17]. Although hyperuricemia is typically closely associated with the generation of ROS and inflammation, studies have found that total antioxidant activity (TAA) in the saliva is significantly increased in obese individuals with hyperuricemia [18]. The main water-soluble antioxidant in saliva is uric acid. Its concentration is strongly correlated with TAA, and uric acid accounts for more than 70% of the total antioxidant activity in saliva [19].

Saliva is an ideal sample for studying the oral microbiome as it reflects the flora status of the entire oral microenvironment [20]. As one of the key sites included in the Human Microbiome Project (mouth, nasal, vaginal, gastrointestinal, and skin) [21], saliva can influence systemic diseases. This occurs through direct pathways that move to distant sites and indirect effects from microbial imbalance [21]. For example, in the OSA group, *Rothia* and *Gemella* were significantly more abundant, while *Streptococcus* and *Veillonella* were significantly decreased compared to the non-OSA group [22]. The oropharyngeal microbiota in middle-aged OSA patients exhibited significantly reduced species diversity and abundance [23]. Liu *et al*. reported that patients with HUA exhibited significantly higher salivary intermediate *Prevotella* levels but significantly lower *Serratia marcescens* levels compared to healthy controls [24].

Although previous studies have examined the relationships between OSA and the microbiome, and between HUA and the microbiome separately, the oral microbiome in individuals with comorbid OSA and HUA remains unexplored. Therefore, this study aims to investigate the associations between uric acid and both the oral microbiome and salivary proteins in adult patients with OSA, as well as to explore the underlying mechanisms, with the goal of identifying potential diagnostic and therapeutic strategies for patients with OSA comorbid with hyperuricemia.

## Materials and Methods

### Study Population

This cross-sectional study was conducted in accordance with the Declaration of Helsinki and local regulatory guidelines, and received approval from the Institutional Review Board of Peking University School of Stomatology (PKUSSIRB-201950155). All participants signed the informed consent before being included in the study.

The sample size for the salivary microbiome analysis was estimated using the distance-matching and permutational multivariate analysis of variance (PERMANOVA) method proposed by Kelly *et al*. [25], implemented using the “Micropower” R package. Distance matrices were constructed based on oral microbiome data from the Human Microbiome Project [26] to evaluate effect size (ω²) and statistical power. A smaller ω² indicates greater discriminatory capacity. Power levels of 80% and 90% were assessed across varying group sizes (Table S1). At 90% power and a significance level of *p* = 0.05, an effect size of ω² = 0.027 was achievable with 20 subjects per group, which is smaller than the effect size observed in previous studies investigating antibiotic-associated changes using unweighted Jaccard distance. Therefore, we consider that a group size of 20 participants is sufficient to ensure adequate statistical power.

The sample size for salivary proteomics was calculated based on the statistical power analysis method proposed by Levin [27], using the "pwr" R package. Previous studies have indicated that salivary samples exhibit a coefficient of variation (CV) below 20% [28]. In this study, a significance level of *p* = 0.05, a fold change of 1.5, and a CV of 20%were used for the calculation. The results showed that a statistical power of 90% could be achieved with 7 samples per group. Therefore, a minimum of 7 samples per group was included to ensure that the target power was maintained.

The study consecutively enrolled adult patients who visited the Sleep Medicine Center of Peking University Peoplés Hospital for suspected obstructive sleep apnea and were scheduled for overnight polysomnography between March 2023 and January 2024. The criteria for inclusion were as follows: 1) voluntary participation in the study; 2) age ≥ 18 years, 3) no severe cardiovascular, cerebrovascular, respiratory, or endocrine diseases, malignant or benign tumors, or mental illnesses, and 4) no antibiotic or hormone treatment in the past 3 months. The criteria for exclusion were as follows: 1) diagnosis of other types of sleep disorders (narcolepsy, restless legs syndrome, etc.), 2) presence of defective dental restorations or severe periodontal disease, caries, or mucosal diseases in the oral cavity, especially immunological conditions, 3) receipt of periodontal treatment other than periodontal scaling within the past 6 months, 4) smokers (including those who quit less than 6 months ago) and participants with alcohol use disorders, 5) participants with a limited dietary structure, and 6) receipt of treatment for obstructive sleep apnea in the past 6 months.

### Clinical and Laboratory Assessment

In this study, all participants underwent overnight polysomnography and participated in a comprehensive collection of medical histories, which included anthropometric measurements, fasting blood sampling, and clinical assessments. Sleep profiles, including quality, daytime drowsiness, and insomnia, were assessed by the Pittsburgh Sleep Quality Index (PSQI), Epworth Sleepiness Scale (ESS), and Insomnia Severity Index (ISI).

The diagnosis of OSA was determined using guidelines established by the American Academy of Sleep Medicine, with the apnea-hypopnea index (AHI) of 5 or more events per h [29]. Meanwhile, hyperuricemia was characterized as having fasting serum urate concentrations surpassing 420 μmol/l in males and 360 μmol/l in females [30]. Serum uric acid was measured using standard clinical biochemical assays. Following an overnight fast of at least 8 hours, venous blood samples were collected in the morning and analyzed by the clinical laboratory of Peking University Peoplés Hospital.

Based on the presence of OSA and hyperuricemia, participants were first classified into three groups (Table 1): those with OSA and hyperuricemia (OSA+HUA group), those with isolated OSA (OSA group), and non-OSA healthy controls (Control group). To further assess the relationship between changes in uric acid levels and the microbiome, we included OSA patients who were on medication and had well-controlled uric acid levels, and named this group OSA+CHUA (Table 1).

### Saliva Sample Collection

In accordance with the Human Microbiome Project’s oral microbiome sampling protocol [31], participants were instructed to collect 2 ml of unstimulated saliva upon waking between 6:00 am and 8:00 am. They were required to fast for at least 2 h prior to sampling and avoid brushing their teeth or using any oral hygiene products. During collection, participants were advised to tilt their heads down in a quiet state to allow saliva to naturally accumulate in the mouth, which can then flow from the corner of the mouth into a sterile 15 ml centrifuge tube. The samples were kept on ice and delivered to the lab within an 1 h. After centrifugation at 12,000 ×*g* for 30 min at 4°C, the pellets and supernatants were transferred to separate 1.5 ml tubes and preserved at -80°C.

### DNA Extraction and 16S rRNA Gene Sequencing

The DNA of the salivary microbiota was extracted by the MagBeads FastDNA Kit for Soil (116564384, MP Biomedicals, USA). The DNA concentration was measured with a nanodrop spectrophotometer (NC2000, Thermo Fisher Scientific, USA), while its quality was assessed using agarose gel electrophoresis.

Polymerase chain reaction (PCR) amplification targeted the V3–V4 region of the 16S rRNA gene. The PCR reaction mixture included 5 μl of 5× reaction buffer and high GC buffer each, 2 μl of dNTPs (10 mM), 0.25 μl of Q5 high-fidelity DNA polymerase, 2 μl of DNA template, 1 μl of each forward and reverse primer (10 μM), and 8.75 μl of ddH_2_O. The PCR protocol began with initial denaturation at 98°C for 5 min, followed by 25 cycles of denaturation (98°C, 30 sec), annealing (52°C, 30 sec), and extension (72°C, 45 sec). A final extension phase at 72°C for 5 min concluded the PCR run, with samples subsequently stored at 12°C. After selecting the target fragments from the amplification products, purification and recovery were performed using the magnetic bead separation method.

The Quant-iT PicoGreen dsDNA assay kit (P7589, Invitrogen, USA) was utilized to quantify the PCR products on a BioTek FLx800 microplate reader. Samples were pooled based on their sequencing depth requirements. Subsequently, libraries were prepared, and those meeting quality standards underwent paired-end sequencing (2 × 250 bp) on the NovaSeq 6000 high-throughput sequencing platform (Illumina, USA), utilizing the NovaSeq 6000 SP Reagent Kit (500 cycles, Illumina).

The sequencing data underwent analysis through the Divisive Amplicon Denoising Algorithm 2 (DADA2) plugin [32]. The analysis included quality filtering, denoising, merging, and chimera removal, culminating in the generation of amplicon sequence variants (ASVs). The ASVs were subsequently classified using the Human Oral Microbiome Database [33].

### Protein Extraction and Astral DIA Proteomic Analysis

Proteins from each sample were extracted with an SDT lysis buffer (4% SDS, 100 mM Tris-HCl, pH 7.6) and quantified using the BCA Protein Assay Kit (P0012, Beyotime, China). The quality of the extracted proteins was evaluated through SDS-PAGE alongside Coomassie Brilliant Blue R-250 staining. A mixed sample, containing 15 μg of protein from each, was prepared for database construction and quality control. All samples, including the pooled sample, underwent trypsin digestion by filter-aided sample preparation [34]. After being desalted with C18 Cartridges (Empore SPE Cartridges MCX, 30 μm, Waters), the resulting peptides were lyophilized and reconstituted in 0.1% formic acid (40 μl). Peptide concentrations were estimated by UV absorbance at 280 nm. Each sample was supplemented with indexed retention time (iRT) calibration peptides to aid in data-independent acquisition (DIA) analysis. A Vanquish Neo liquid chromatography system (Thermo Fisher Scientific) in DIA mode was used in conjunction with an OrbitrapTM AstralTM mass spectrometer (Thermo Fisher Scientific) to examine peptides. In the first stage of mass spectrometry (MS), precursor ions were scanned over a mass range spanning 380 to 980 m/z, with a resolution of 240,000 at 200 m/z. The process utilized a normalized automatic gain control (AGC) target set at 500%, with an ion injection time (IT) capped at 5 milliseconds. In the second stage of MS, DIA was performed with 299 scanning windows, each featuring a 2 m/z isolation width. Higher-energy collisional dissociation was applied at an energy level of 25 eV, accompanied by the same normalized AGC target of 500% and a reduced maximum IT of 3 milliseconds. DIA-NN version 1.8.1 was used to analyze the generated DIA data. The main parameters included trypsin as the enzyme, allowing for a maximum of one missed cleavage, with carbamidomethyl (C) established as a fixed modification. Additionally, oxidation (M) and acetylation (at the protein N-terminus) were considered as dynamic modifications. A false discovery rate below 1% and a 99%confidence threshold were used for protein identification. Protein identification was further confirmed using the public UniProt database.

### Bioinformatics and Statistical Analysis

Microbial diversity analysis included both alpha-diversity and beta-diversity assessments. Alpha-diversity was assessed to evaluate species’ richness, diversity and evenness, including the Chao1, observed species and Faith’s phylogenetic diversity (Faith’s PD). Beta-diversity, reflecting variations in species composition across communities, was analyzed using the diversity core-metrics algorithm based on Jaccard distance. Principal coordinate analysis (PCoA) was used to visualize the data in a two-dimensional plot, and statistical differences between groups were tested with PERMANOVA. Group variances were compared using permutational analyses of multivariate dispersions (PERMDISP). Differential taxa were identified through linear discriminant analysis (LDA) effect size (LEfSe) with an LDA threshold set at 2. The results were displayed using LDA score bar plots and taxonomic cladograms.

Differentially expressed proteins were identified using a fold change threshold of > 1.5 for upregulation or < 0.67 for downregulation, with only those remaining significant differences after Benjamini-Hochberg (BH) correction selected. Homologous sequences were identified by locally searching the selected protein sequences with NCBI BLAST+ (ncbi-blast-2.2.28+-win32.exe) and InterProScan. GO term mapping and sequence annotation were conducted using Blast2GO, and the GO annotation outcomes were graphically represented with R 4.3.0 (Austria).

Clinical indicators were analyzed using R 4.3.0 (Austria). A two-tailed test with a significance level of *p* < 0.05 was applied unless stated otherwise. To assess normality, continuous variables were subjected to the Shapiro-Wilk test. Data that met the normal distribution criteria were reported as mean ± standard deviation and analyzed using an independent *t*-test for two groups or one-way ANOVA for multiple groups. In contrast, non-normally distributed data were presented as median (with quartiles) and analyzed with the Mann-Whitney U test for two-groups or the Kruskal-Wallis H test for multiple groups. Categorical variables were summarized as frequencies (percentages) and assessed by the chi-square test.

All multi-group comparisons, including pairwise comparisons, were corrected for multiple testing using the BH method to control the false discovery rate. Following correction, *p*-values less than 0.05 were considered statistically significant.

If there are statistical differences in demographic characteristics such as age, BMI and sex, microbiome multivariable associations with linear (MaAsLin) models will be assessed using the MaAsLin 2 package in R [35]. This analysis will help ensure that the observed differences are primarily due to the grouping rather than confounding factors.

## Results

### Description of Participants

A total of 65 participants were recruited, including 23 OSA patients, 22 OSA+HUA patients, and 20 controls. Table 1 presents the demographic and clinical attributes for each group. There were no notable differences observed in demographic characteristics, blood pressure, lowest peripheral oxygen saturation (LSpO_2_), and AHI between the OSA and OSA+HUA groups.

### Diversity Analysis of Salivary Microbiome

The analysis of 65 samples yielded a total of 6,132,694 raw reads. After filtering and denoising, 5,203,416 high-quality reads remained. On average, each sample yielded 80,053 reads for analysis.

**Fig. 1:**
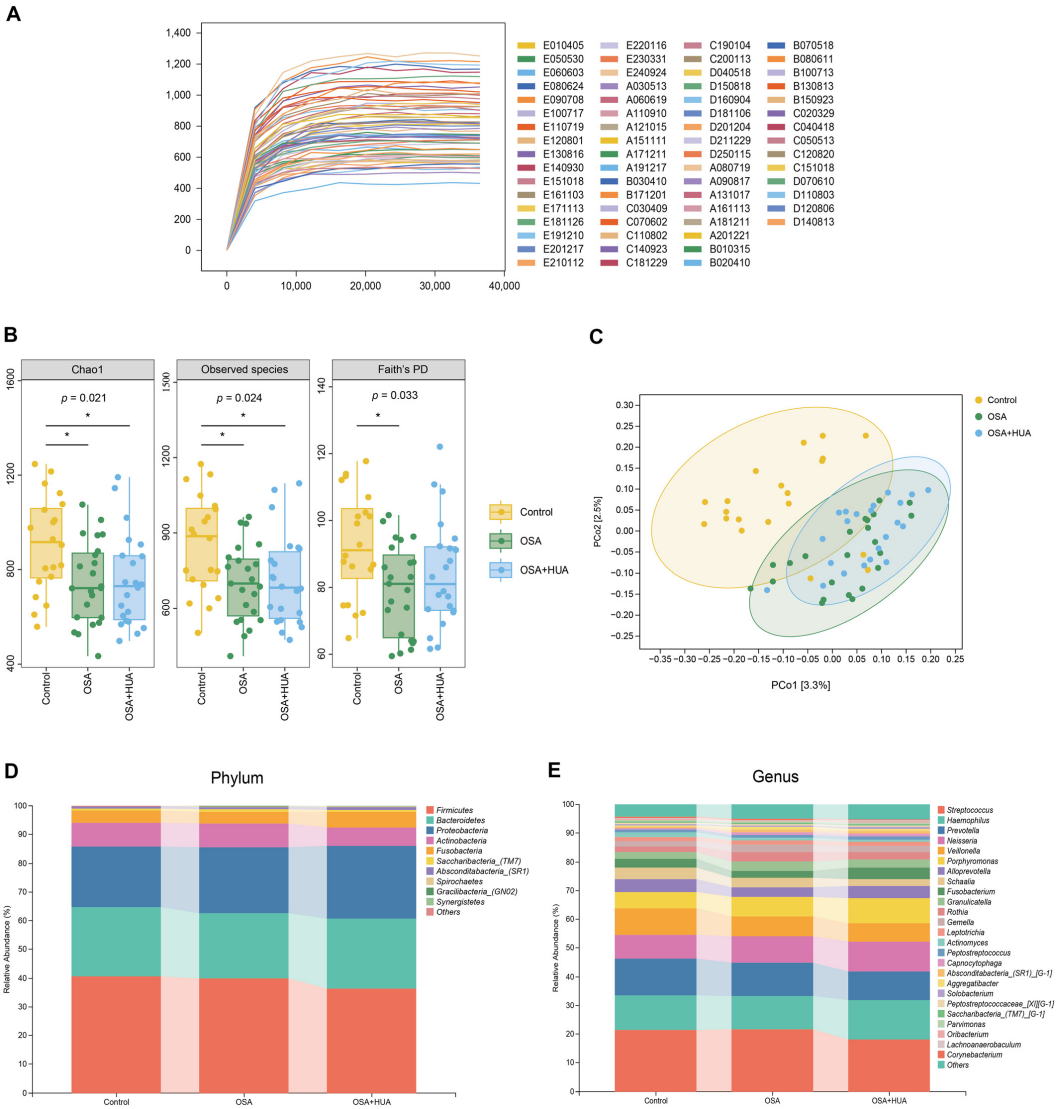
Salivary microbiome characteristics of OSA, OSA with comorbid HUA, and control. (**A**) Rarefaction curves for each sample, with sampling depth on the x-axis and the median alpha-diversity index calculated over 10 iterations on the y-axis. (**B**) Alpha-diversity within each group, assessed using Chao1, Observed species, and Faith's PD indices. (**C**) PCoA of beta-diversity for each group, based on Jaccard distance (composition-based). (**D**) Top 10 phylum-level microbial compositions and (**E**) the top 25 genera for each group, indicating relative abundance. Asterisks (*, ** and ***) denote statistical significance at *p* < 0.05, *p* < 0.01, and *p* < 0.001, respectively.

The rarefaction curve reached a plateau, indicating adequate sequencing depth across all samples (Fig. 1A). In alpha-diversity, the Chao1 and observed species indices were lower in the OSA and OSA+HUA groups compared with the control group. The Faith’s PD index demonstrated a significant reduction in the OSA group compared to the controls, with no notable difference between OSA+HUA and control groups (Fig. 1B). The PCoA plot based on beta-diversity (Jaccard distance) showed a trend of separation among groups, indicating differences in species composition across groups (Fig. 1C). PERMANOVA analysis tested intergroup differences, revealing statistically significant results (Control vs. OSA, *p* = 0.001; Control vs. OSA+HUA, *p* = 0.001; OSA vs. OSA+HUA, *p* = 0.034). Additionally, PERMDISP was applied to assess variance within groups, showing no statistically significant differences in group dispersions (Control vs. OSA, *p* = 0.705; Control vs. OSA+HUA, *p* = 0.844; OSA vs. OSA+HUA, *p* = 0.864).

### Composition of Microbial Taxa in the Salivary Microbiome

Fig. 1D shows the top 10 phyla by relative abundance, with *Firmicutes*, *Bacteroidetes*, and *Proteobacteria* representing the three dominant components of the salivary microbiome, each exceeding 20% in relative abundance. Fig. 1E shows the top 25 genera by relative abundance, with *Streptococcus*, *Haemophilus*, *Prevotella*, *Neisseria*, and *Veillonella* as the most dominant genera. Compared to the control and OSA groups, the OSA+HUA group exhibited a higher relative abundance of *Haemophilus*, *Neisseria*, *Porphyromonas*, *Fusobacterium*, and *Leptotrichia*. In contrast, genera with lower relative abundance in the OSA+HUA group included *Streptococcus*, *Prevotella*, *Veillonella*, *Schaalia*, and *Actinomyces*.

### Comparison of Microbiota among Different Groups

**Fig. 2:**
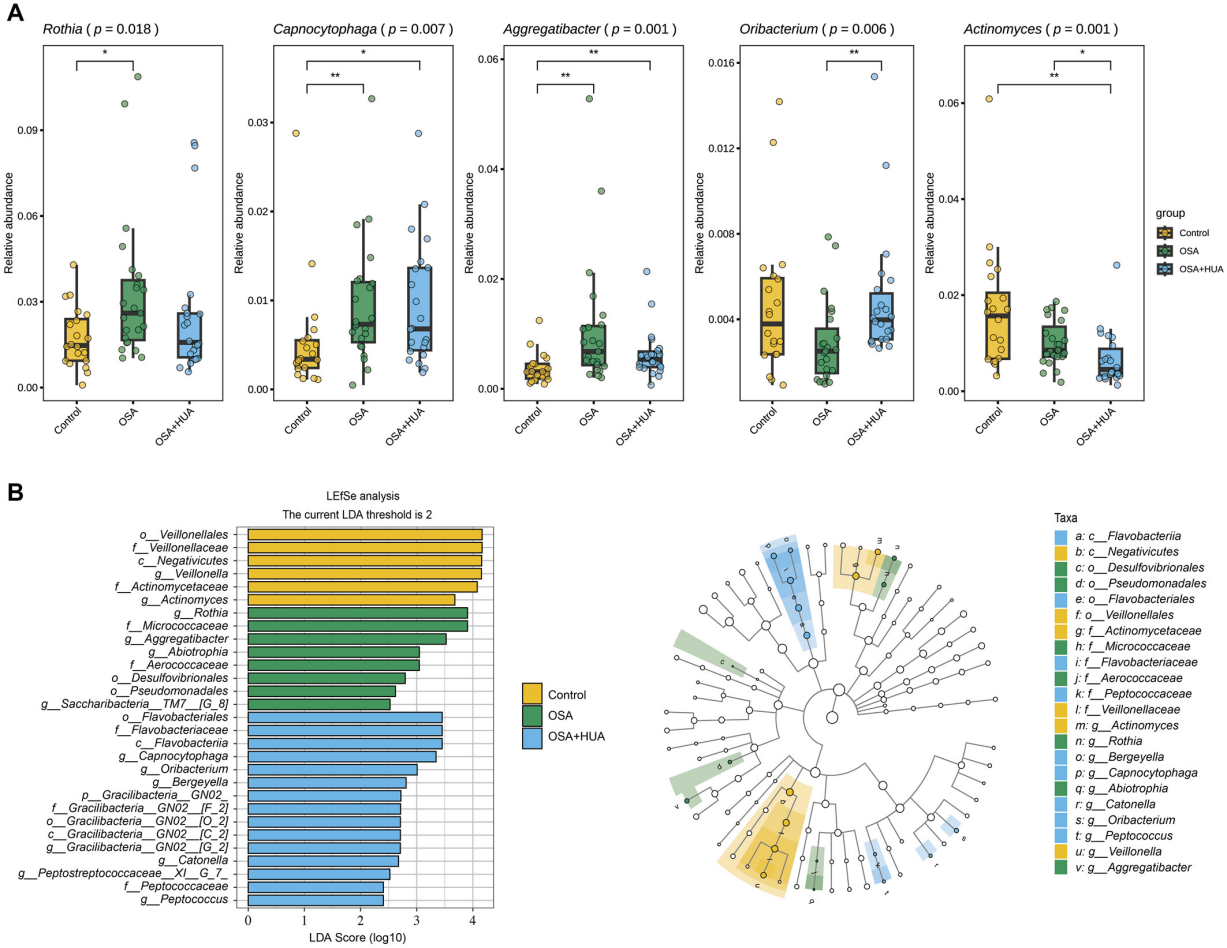
Differential genera of OSA, OSA with comorbid HUA, and control. (**A**) Differential genera identified among the top 25 genera by relative abundance. (**B**) The composition of microbiota was examined through LEfSe, with only LDA scores exceeding 2 included. The histogram displays significant taxonomic differences among the Control, OSA, and OSA+HUA groups, with longer bars indicating greater significance. The cladogram illustrates the hierarchical relationships of taxa from the phylum to species level, with colored nodes representing enriched taxa in each group. Asterisks (* and **) denote statistical significance at *p* < 0.05 and *p* < 0.01, respectively.

We identified 5 differential genera among the top 25 genera in terms of relative abundance (Fig. 2A). The OSA group exhibited significantly higher levels of *Rothia* (*p* = 0.019), *Capnocytophaga* (*p* = 0.009), and *Aggregatibacter* (*p* = 0.001) compared to the controls. Furthermore, compared to the OSA group, the OSA+HUA group showed a notable rise in *Oribacterium* abundance (*p* = 0.002), while the abundance of *Actinomyces* further decreased (OSA vs. OSA+HUA: *p* = 0.012; Control vs. OSA+HUA: *p* = 0.001). Additionally, in the OSA+HUA group, the abundances of *Capnocytophaga* (*p* = 0.015) and *Aggregatibacter* (*p* = 0.009) were significantly elevated compared to the control group.

To further identify the key differential genera among the groups, we performed differential analysis at multiple taxonomic levels using LEfSe. The analysis showed that six taxa were abundant in the Control group, eight taxa were enriched in the OSA group, and fifteen taxa were significantly elevated in the OSA+HUA group (Fig. 2B).

In this study, the distinct color variations among groups prominently emphasize the notable intergroup differences. At the genus level, the OSA group showed marked enrichment in *Rothia*, *Aggregatibacter*, and *Abiotrophia*, while the OSA+HUA group showed significant enrichment in *Capnocytophaga*, *Oribacterium*, and *Bergeyella*. In the Control group, *Veillonella* and *Actinomyces* were significantly enriched.

### Validation of Oral Microbiota Dysbiosis with New Subgroup

To further investigate the association between changes in uric acid levels and oral microbiota, we included seven OSA patients who had normalized their uric acid levels through medication, forming a new subgroup named OSA+CHUA. General information for the different groups is presented in Table 1. There were no significant differences in demographic characteristics, blood pressure, LSpO_2_, and AHI among the non-control groups.

**Fig. 3:**
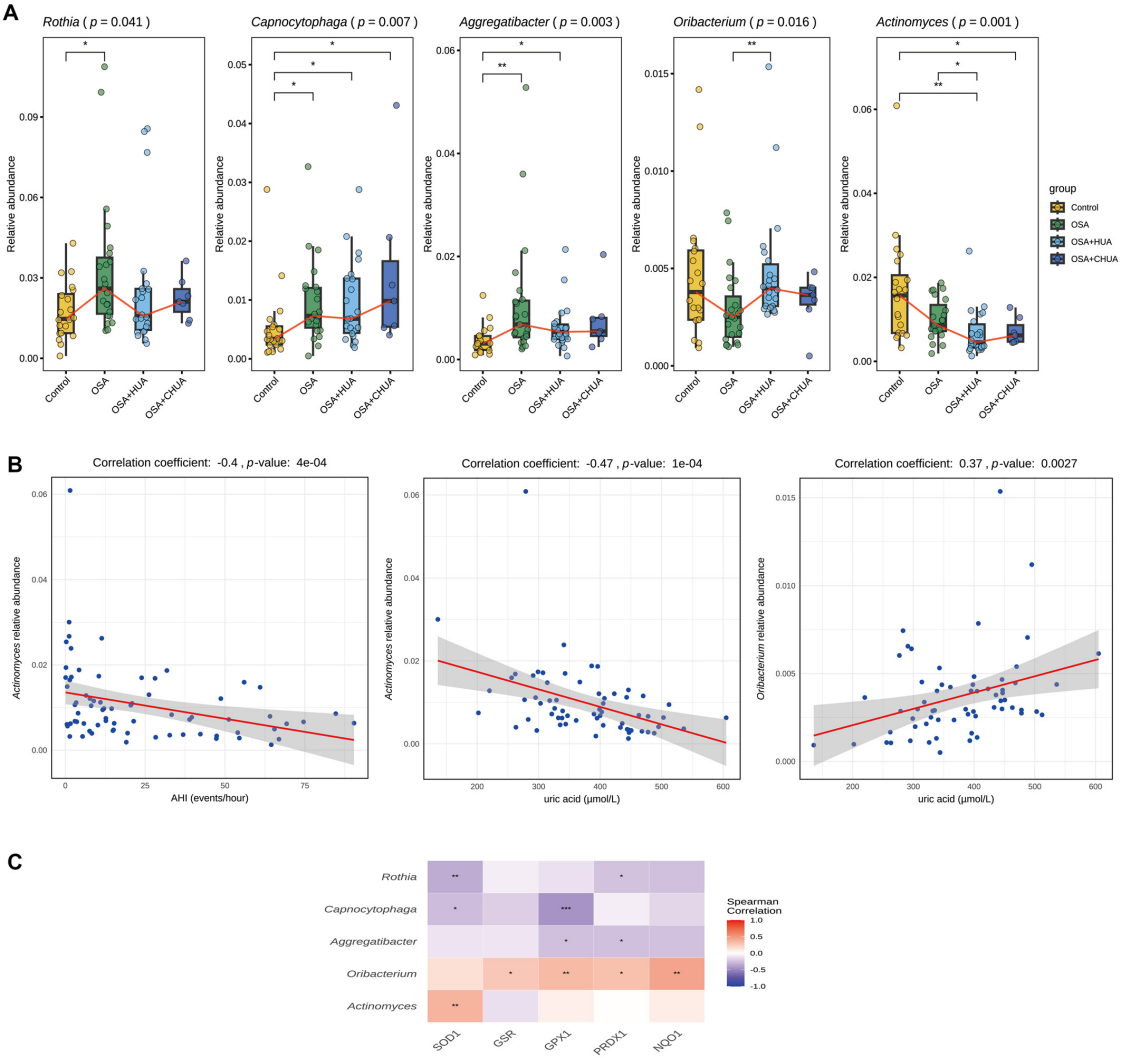
Differential genera abundance and its associations with AHI, uric acid, and antioxidant proteins across four groups. (**A**) Relative abundance changes of differential genera across the four groups. The red line represents the median trend line. (**B**) Spearman correlation analysis between the apneahypopnea index and *Actinomyces*, and between uric acid and *Actinomyces* and *Oribacterium*. (**C**) Correlation analysis between the antioxidant proteins and differential genera. Asterisks (*, ** and ***) denote statistical significance at *p* < 0.05, *p* < 0.01, and *p* < 0.001, respectively.

We further analyzed the changes in differential genera across groups and observed a fluctuating pattern of microbial abundance following hyperuricemia treatment (Fig. 3A). Compared with the control group, *Rothia*, *Capnocytophaga*, and *Aggregatibacter* showed increased abundance in the OSA group, and this abundance slightly decreased in the OSA+HUA group. However, this decrease was partially reversed after hyperuricemia was medically controlled. Conversely, *Oribacterium* displayed the opposite trend, with reduced abundance in the OSA group compared to the control group, an increase in the OSA+HUA group, and a subsequent decline after hyperuricemia treatment. The changes in *Actinomyces* were distinct, showing a decrease in abundance in the OSA group, a further reduction in the OSA+HUA group, and a reversal trend after hyperuricemia treatment. Spearman correlation analysis revealed that *Actinomyces* was significantly negatively correlated with AHI and uric acid levels, whereas *Oribacterium* was significantly positively correlated with uric acid levels (Fig. 3B). In addition, MaAsLin analysis revealed that none of the differential genera showed significant associations with age, BMI, or sex (Table S2).

Cu/Zn superoxide dismutase (SOD1), glutathione reductase (GSR), glutathione peroxidase 1 (GPX1), peroxiredoxin-1 (PRDX1) and NAD(P)H quinone dehydrogenase 1 (NQO1) are well-known antioxidant proteins [36-38]. Spearman correlation analysis between the differential genera and these proteins revealed that the abundances of *Rothia*, *Capnocytophaga*, and *Aggregatibacter* were generally negatively correlated with the expression of several antioxidant proteins. In contrast, *Oribacterium* showed a positive correlation with the expression of several antioxidant proteins, while the relationship between *Actinomyces* and these proteins remained unclear (Fig. 3C).

### Differentially Expressed Proteins between OSA and OSA+HUA Groups

**Fig. 4:**
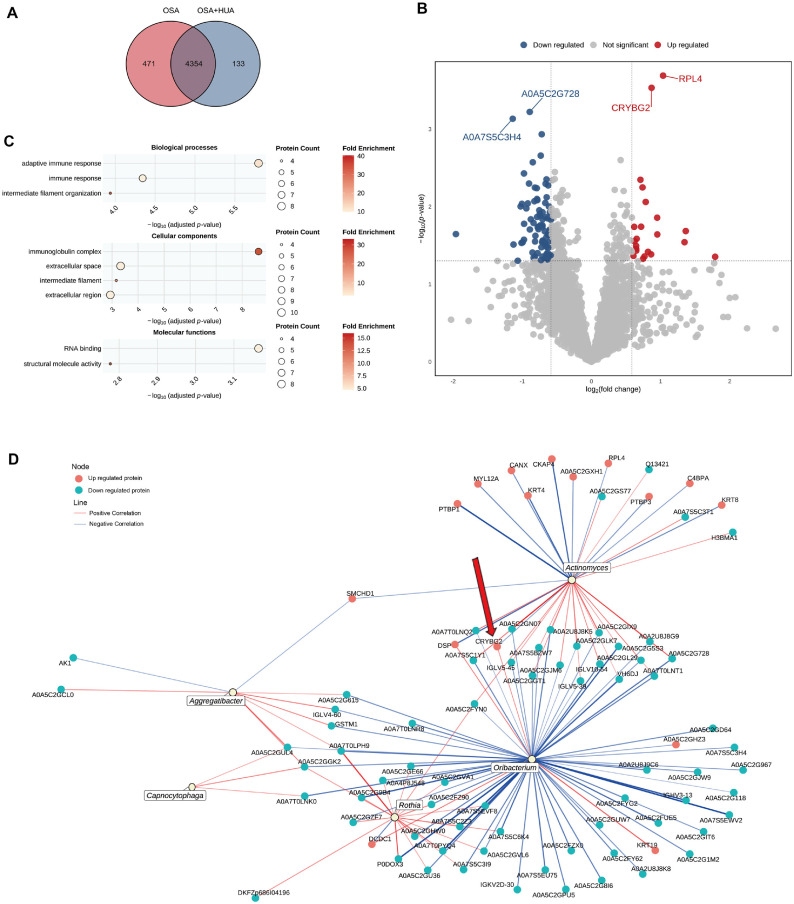
The function of differentially expressed proteins and their correlation with differential genera. (**A**) Venn diagram showing common and distinct proteins. (**B**) Volcano plot displaying upregulated (red) and downregulated (blue) proteins (*p* < 0.05, fold change > 1.5 or < 0.67), with proteins *p* < 0.001 labeled. The plot uses unadjusted *p*-values, but only shows proteins that remain significant after Benjamini-Hochberg (**BH**) correction. (**C**) GO enrichment analysis bubble charts depicting the GO terms enriched by differentially expressed proteins in biological processes, cellular components, and molecular functions, respectively. The size of each bubble reflects the number of proteins involved in the enrichment, while the color indicates the magnitude of the enrichment fold. The darker the color, the higher the fold enrichment in the GO term. (**D**) The network diagram illustrates the relationships between differentially expressed proteins and differential genera. Orange-red circles represent upregulated differential proteins, while blue-green circles represent downregulated differential proteins. Red lines indicate positive correlations, while blue lines indicate negative correlations. The thickness of the lines corresponds to the strength of the correlation.

Saliva proteins from 23 OSA patients and 22 OSA+HUA patients were included in the analysis. A total of 4,958 proteins were identified, with a Venn diagram showing 471 proteins unique to the OSA group and 133 proteins unique to the OSA+HUA group (Fig. 4A). Based on the selection criteria (*p* < 0.05, fold change > 1.5 or < 0.67), 104 differentially expressed proteins were identified between the two groups, with 22 proteins upregulated and 82 proteins downregulated in the OSA+HUA group compared to the OSA group. Further selection of proteins with a *p*-value < 0.001 revealed that large ribosomal subunit protein uL4 (RPL4) and beta/gamma crystallin domain-containing protein 2 (CRYBG2) were significantly upregulated, while the unreviewed proteins A0A5C2G728 and A0A7S5C3H4 were significantly downregulated in the OSA+HUA group compared to the OSA group (Fig. 4B). GO enrichment analysis showed that the main components include those related to immunity, such as adaptive immune response and immunoglobulin complex, as well as those related to cell structure and function, such as intermediate filament organization, intermediate filament, extracellular space and extracellular region (Fig. 4C).

### Correlation between Differentially Expressed Proteins and Genera across All Groups

A correlation analysis was performed between the differential expressed proteins and differential genera (Fig. 4D). *Oribacterium* is the genus with the most correlations with differential expressed proteins among all the differential genera. Specifically, *Oribacterium* exhibited a positive correlation with most upregulated differential proteins and a negative correlation with most downregulated differential proteins, which is consistent with the observed increase in *Oribacterium* abundance in the OSA+HUA group. Furthermore, we found that *Oribacterium* was significantly positively correlated with the CRYBG2 protein (correlation coefficient r = 0.293, *p*-value = 0.022), as indicated by the red arrow. Compared to the OSA group, both CRYBG2 and *Oribacterium* expression levels were significantly upregulated in the OSA+HUA group.

## Discussion

OSA is a heterogeneous disorder with complex pathophysiological mechanisms that can affect multiple systemic functions. A primary focus in OSA research is classifying OSA comorbidities into different types. HUA is a common complication in OSA. However, patients with this type often do not show typical OSA symptoms, such as increased daytime sleepiness. Identifying biomarkers to distinguish OSA subtypes is essential. This could help to improve the management of OSA-related clinical manifestations and comorbidities. Previous studies have explored the correlation between the microbiome and each of OSA and HUA, but no study has explored the microbiome of patients with comorbid HUA with OSA. This is the first study to employ high-throughput sequencing to investigate the salivary microbiota and protein of Chinese adults with comorbid OSA and HUA.

OSA patients are usually associated with a state of high oxidative stress [3]. Uric acid is considered a strong antioxidant [15, 16]. It may help reduce this oxidative stress. Our research found that RPL4 showed a significant increase in the OSA+HUA group relative to the OSA group. Animal studies have shown that the regulation of RPL4 can enhance antioxidant stress capacity [39]. CASIN (a specific inhibitor of Cdc42 activity) can exert anti-aging effects on the skin of naturally aging mice through RPL4, exhibiting anti-inflammatory properties while influencing collagen synthesis and cell cytoskeleton morphology, and effectively reducing reactive oxygen species levels [40]. The second most upregulated protein was CRYBG2. Although research on CRYBG2 is relatively limited, several studies have suggested that beta/gamma crystallins have antioxidant effects. Gamma-crystallin acts as a redox enzyme, involved in protein changes under oxidative stress conditions [41]. Supplementation with selenite and ebselen can alleviate oxidative damage in rat lenses and promote the expression of beta-crystallin [42]. Both A0A5C2G728 and A0A7S5C3H4 are significantly downregulated differential proteins. Specifically, A0A7S5C3H4 is associated with the immunoglobulin complex and adaptive immune response. In GO enrichment analysis, the differentially expressed proteins are closely associated with immune function and intermediate filament organization, which may relate to enhancing cellular antioxidant capacity by regulating immune responses and modulating barrier functions to alleviate oxidative stress.

Regarding microbiome alterations, the alpha-diversity in the OSA and OSA+HUA groups was significantly reduced compared to the control group, aligning with findings from other studies [23, 43]. Additionally, in this study, we found a significant increase in the abundance of *Rothia*, *Capnocytophaga*, and *Aggregatibacter* in the OSA group compared to the control group. This finding aligns with our prior research as well as other studies [44]. In both our 2021 and 2022 studies, we found a significantly higher abundance of *Rothia* in the OSA group relative to the control group [22, 45]. *Rothia* species are predominantly facultative anaerobes [46, 47], and previous studies have shown a significant positive correlation between *Rothia* abundance and oxidative stress levels, as well as pro-inflammatory factors like cyclooxygenase-2, interleukin-6, and tumor necrosis factor-α (TNF-α) [48]. It has been shown that *Rothia dentocariosa* induces TNF-α production in the host through a toll-like receptor 2 dependent mechanism [49]. An animal study further revealed a negative correlation between the relative abundance of *Rothia* and serum antioxidant enzyme activities [50]. In our study, we also found that *Rothia* was significantly negatively correlated with several antioxidant proteins. This may explain why *Rothia* is enriched in the salivary microbiome of OSA patients, while its abundance decreases when OSA comorbid HUA and rebounds after HUA treatment.

*Capnocytophaga* and *Aggregatibacter* exhibited similar trends to *Rothia*. We observed significant enrichment of *Aggregatibacter* and *Capnocytophaga* in the OSA group. Gao *et al*. reported that *Capnocytophaga* was significantly enriched in severe OSA patients compared to those who underwent continuous positive airway pressure (CPAP) treatment [51]. In our study, *Capnocytophaga* abundance was significantly elevated in the OSA group compared to controls, although we did not include post-treatment OSA patients. Leadbetter *et al*. noted that *Capnocytophaga* is a facultative anaerobe that depends on carbon dioxide for growth, aligning with OSA characteristics such as intermittent hypoxia and hypercapnia [52]. Additionally, studies have suggested that *Capnocytophaga*’s tolerance to oxidative stress is associated with its ability to respond to peroxide-sensing transcriptional regulators, including OxyR [53]. OxyR, a recognized peroxide-sensing transcriptional activator, regulates antioxidant enzymes and ROS scavengers [54], enabling *Capnocytophaga* to adapt to oxidative stress environments. *Aggregatibacter*, closely associated with periodontitis [55], stimulates the host immune system and induces inflammatory responses by producing virulence factors such as leukotoxin and lipopolysaccharide. These factors stimulate the immune system and trigger the release of pro-inflammatory cytokines like interleukin-1β and TNF-α. Moreover, *Aggregatibacter* promotes the oxidation of low-density lipoprotein through mechanisms involving oxidative stress, including reactive oxygen species generated by nicotinamide adenine dinucleotide phosphate (NADPH) oxidase and myeloperoxidase [56].

Additionally, our study revealed a significant decrease in the abundance of *Actinomyces* in the OSA+HUA group compared to controls. This decrease could be reversed with hyperuricemia medication control, and spearman correlation analysis indicated a significant negative association between *Actinomyces* abundance and uric acid levels. However, we found no supporting studies for this observation. Some research suggests a negative correlation between *Actinomyces* and antioxidant enzyme activity [57]. However, in our study, the relationship between *Actinomyces* and antioxidant proteins was unclear. Dunning *et al*. reported that certain *Actinomyces* species are moderately sensitive to oxidative damage caused by Fe^2+^ under strictly anaerobic conditions, even at uric acid concentrations of 10 mM, which fail to protect them [58]. This may explain the changes in *Actinomyces* abundance observed in our study. Besides, we did not observe significant differences in *Actinomyces* abundance between the control and OSA groups, consistent with the findings of Jia *et al*. [59].

Furthermore, *Oribacterium* abundance was significantly higher in the OSA+HUA group compared to the OSA group, with a significant positive correlation between *Oribacterium* abundance and uric acid levels. Liu *et al*. identified a significant association between the salivary abundance of two unclassified *Oribacterium* species and serum uric acid levels in a study of 1,915 individuals [60]. Gem *et al*. also found that *Oribacterium asaccharolyticum* was associated with higher uric acid levels in males, suggesting a potential mechanism involving the antioxidant activity of uric acid, consistent with our findings [61]. In our study, we found that *Oribacterium* was significantly positively correlated with several antioxidant proteins, and it was also significantly positively correlated with CRYBG2, which was significantly upregulated in our study, a member of the beta/gamma crystallin protein family with potential antioxidant functions. Additionally, a rat study demonstrated that *Oribacterium* abundance recovered after inflammatory states were alleviated by suppressing pro-inflammatory cytokines [62]. We hypothesize that *Oribacterium* is sensitive to oxidative stress, with OSA-induced hypoxemia increasing oxidative stress levels and reducing *Oribacterium* abundance. This process can be reversed by uric acid’s antioxidant activity. Thus, *Oribacterium* may serve as a potential biomarker for hyperuricemia.

This study has some limitations. Patients were grouped based on suspected OSA when they came to the hospital. OSA is more common in middle-aged and older men. As a result, the control group had different age and gender characteristics compared to the OSA groups. Future studies should match these factors better. Additionally, the study primarily includes male Chinese individuals, and whether these findings can be generalized to other populations worldwide remains uncertain. Furthermore, the causal relationship between salivary microbiota and OSA needs further validation. This can be done through CPAP treatment interventions or in vivo experiments. Moreover, directly measuring purine metabolism intermediates (such as hypoxanthine and xanthine) and oxidative stress markers (such as ROS levels and lipid peroxidation products) could provide deeper insights into the underlying mechanisms of change. Lastly, the sample size of the OSA+CHUA group in this study is limited, and future research should aim to expand the sample size to achieve more robust and generalizable results.

## Supplemental Materials

Supplementary data for this paper are available on-line only at http://jmb.or.kr.



## Figures and Tables

**Table 1:** Demographic and clinical characteristics of the study participants.