# Supplementary material for: Association between Serum Uric Acid Levels and Salivary Microbiota in Patients with Obstructive Sleep Apnea
Source: J Microbiol Biotechnol. 2025 Jun 23;35:e2503042. doi: 10.4014/jmb.2503.03042 (PMC12256837; doi:10.4014/jmb.2503.03042)
Supplement: Supplementary file 1 [file jmb-35-e2503042-supple.pdf]

J. Microbiol. Biotechnol. 2025. 35: e2503042
https://doi.org/10.4014/jmb.2503.03042

Supplementary Tables

Table S1. Effect sizes (ω^2) for PERMANOVA with 80% and 90% power at different sample sizes.

Sample size	ω^2	
	80% power	90% power
5	0.0766	0.092
10	0.0311	0.0411
20	0.0202	0.0269
30	0.00907	0.013
40	0.00645	0.0105

The distance matrix simulation was performed using the Human Microbiome Project dataset. The "Micropower" package (http://github.com/brendankelly/micropower) was utilized to evaluate the effect size and statistical power.

Table S2. MaAsLin2 analysis results of microbial abundance with age and BMI as covariates.

genus	variable	regression coefficient	standard deviation	*q*-value
Actinomyces	Age	-0.033	0.010	0.054
	BMI	-0.010	0.035	0.896
	Sex	-0.028	0.318	0.985
Aggregatibacter	Age	-0.023	0.012	0.258
	BMI	0.030	0.041	0.739
	Sex	-0.562	0.371	0.312
Capnocytophaga	Age	0.005	0.012	0.867
	BMI	0.002	0.042	0.985
	Sex	0.581	0.379	0.312
Oribacterium	Age	-0.020	0.012	0.295
	BMI	0.036	0.042	0.686
	Sex	-0.167	0.376	0.867
Rothia	Age	0.018	0.008	0.138
	BMI	0.035	0.027	0.403
	Sex	0.070	0.239	0.896

For each genus, the regression coefficients represent the direction and magnitude of the association with the respective variable. The standard deviation reflects the precision of the regression coefficient estimates. The *q*-value represents the significance of the results after adjustment for multiple comparisons using the Benjamini-Hochberg method.
